# Effect of Oxygen Vacancy Concentration on the Electrical Properties and Microstructure of *Bi*_4_*Ti*_3_*O*_12_ Ceramics: Experimental and First-Principles Investigation

**DOI:** 10.3390/ma18112666

**Published:** 2025-06-05

**Authors:** Tao Chen, Yang Chen, Ning Zhang, Tiantian Liu, Songlin Wang, Qi Zhang

**Affiliations:** 1 School of Electronic Information and Automation, Tianjin University of Science and Technology, Tianjin 300222, China; chenyang6@mail.tust.edu.cn (Y.C.);; 2 School of Microelectronics, Tianjin University, Tianjin 300072, China

**Keywords:** oxygen vacancy concentration, dielectric loss, VASP calculation, DOS density of states, energy band structure

## Abstract

This paper investigates the impact of sintering temperature on oxygen vacancy concentration and its subsequent effect on the microstructure and electrical properties of $Bi_{4}Ti_{3}O_{12}$ (BIT) ceramics. To further clarify these effects, VASP software was employed to simulate BIT ceramics with varying oxygen vacancy concentrations.The experimental results demonstrate that sintering temperature significantly influences the oxygen vacancy concentration. At the optimal sintering temperature of 1080 °C, the BIT ceramics exhibit a balanced microstructure with a grain size of 4.16 μm, the lowest measured oxygen vacancy concentration of 18.44%, and a piezoelectric coefficient ($d_{33}$) of 9.8 pC/N. Additionally, the dielectric loss (tanδ) remains below 0.2 at 500 °C, indicating excellent thermal stability. VASP-based simulations reveal that increasing the oxygen vacancy concentration from 18.56% to 44.55% results in a significant collapse of the band gap (from 2.8 eV → 1.0 eV) and a transition in conductivity type from p-type to n-type. This shift induces a leakage current-dominated threshold effect, leading to a decrease in piezoelectric properties ($d_{33}$ reduced from 9.8 to 6.9 pC/N). Atomic-scale density of states (DOS) analyses indicate that the delocalization of $Ti^{3+}$ and the weakening of Bi–O hybridization collectively induce lattice distortion and ferroelectric inconsistency. These changes are correlated with an increase in dielectric loss and a slight reduction in Curie temperature (from 620 °C → 618 °C). In conclusion, this study comprehensively elucidates the influence of oxygen vacancy concentration on the microstructure and electrical properties of BIT ceramics. The findings provide a theoretical foundation and practical insights for designing high-performance piezoelectric ceramics.

## 1. Introduction

Piezoelectric materials are widely used in sensors, actuators, ultrasonic devices, and energy recovery systems because of their ability to efficiently convert mechanical energy into electrical energy. The demand for such materials has increased in response to the rapid advancement of technological needs, such as monitoring high-temperature conditions in aerospace engines, nuclear power plants, and thermoelectric energy recovery systems. As a result, research has increasingly focused on developing high-temperature piezoelectric materials that can function stably at temperatures exceeding 500 °C [1].

Although contemporary piezoelectric ceramics with a perovskite structure, such as PZT, exhiBIT superior piezoelectric coefficients (>400 pC/N) [2], their relatively low Curie temperatures (Tc < 350 °C) limit their applicability in high-temperature environments [3,4]. In contrast, bismuth titanate (BIT) ceramics with a bismuth laminar structure (BLSF) have been identified as significant candidates for high-temperature piezoelectric materials due to their high Curie temperature (Tc ≈ 675 °C) and substantial spontaneous polarization ($P_{s}$ ≈ 50 $\mu {C/\mathrm{cm}}^{2}$) [5,6]. Compared with other high-temperature piezoelectric materials, $Bi_{4}Ti_{3}O_{12}$ (BIT) ceramics exhibit distinct performance advantages and promising development potential. Unlike lead-free alkali-metal-based $\left(K,Na\right)NbO_{3}$ (KNN), BIT offers a much higher Curie temperature (>675 °C) and superior thermal stability, making it more suitable for operation in harsh, high-temperature environments. Although KNN shows relatively high piezoelectric coefficients ($d_{33}$ ≈ 50–100 pC/N), its poor thermal stability and challenges in achieving high densification limit its applicability under such conditions. Similarly, $LiNbO_{3}$ single crystals possess an exceptionally high Curie temperature (≈1210 °C) and excellent thermal stability, but their low piezoelectric response, high processing complexity, and elevated material costs restrict their widespread use. In contrast, BIT ceramics combine a stable layered structure, reliable thermal performance, environmental benignity, and ease of ceramic processing, making them strong candidates for high-temperature piezoelectric applications. However, despite these advantages, the piezoelectric properties of BIT ceramics are considered inadequate ($d_{33}$ ≈ 8 pC/N), and they exhiBIT substantial conductivity at elevated temperatures, which limits their practical applications [7,8].

The enhancement of BIT ceramics can primarily be achieved through either elemental doping or process modifications [9]. Recent studies have mainly focused on doping strategies to enhance the electrical properties of BIT ceramics [10]. It has been shown that $Nb_{2}O_{5}$ incorporation can simultaneously reduce the conductivity and dielectric loss of BIT ceramics [11,12]. V and W doping improves the remanent polarization ($P_{r}$ > 40 $\mu {C/\mathrm{cm}}^{2}$) by weakening domain wall pinning. Additionally, substituting Ti sites with W or Nb helps to relieve tetragonal lattice strain and decrease the concentration of oxygen vacancies [13]. Beyond single-element doping, co-doping strategies can provide synergistic benefits. For instance, co-doping with W and Cr in $Bi_{4}Ti_{3}O_{12}$ not only reduces electrical conductivity but also enhances $d_{33}$ [14]. Notably, B-site doping (e.g., Zr) is also effective; J.Y. Chen et al. reported that Zr doping significantly increased Pr from 13 $\mu {C/\mathrm{cm}}^{2}$ to 44.55 $\mu {C/\mathrm{cm}}^{2}$ [15]. However, rather than relying solely on doping, this study explores the effect of oxygen vacancy concentration on the properties of BIT ceramics by optimizing the sintering process. Specifically, the sintering temperature of BIT ceramics is adjusted to regulate the concentration of oxygen vacancies. The electrical properties of BIT ceramics are tuned through controlled oxygen vacancy levels, and VASP software is employed to analyze their effects from a fundamental perspective.

## 2. Materials and Methods

The BIT ceramics were prepared using the conventional solid-state reaction method. High-purity $Bi_{2}O_{3}$ (99.90%) and $TiO_{2}$ (99.90%) were used as starting materials. These materials were dried for 6 h and weighed according to stoichiometric ratios. The mixed powders were ball-milled for 24 h at 30 Hz with a ball-to-powder-to-water ratio of 5:1:3, followed by drying for 8 h. The dried powder was calcined at 850 °C to promote phase formation. The synthesized powder then underwent a second ball-milling process under identical conditions, followed by an additional 8-hour drying step. The dried powder was mixed with 5–10 wt% polyvinyl alcohol (PVA) binder for granulation. The granulated powder was uniaxially pressed into 15 mm diameter pellets under 2 MPa for 30 s using a dry press. The green bodies were debindered at 800 °C in a debinding furnace. Subsequently, the samples were sintered at various temperatures (1040 °C, 1060 °C, 1080 °C, and 1100 °C). The sintered ceramics were cut into 0.4 mm thick slices and coated with silver paste via screen printing. After drying, the silver electrodes were fired at 600 °C. Finally, the silver-coated ceramic slices were polarized at 160 °C under an electric field of 5 kV/mm for 1800 s using a polarization system.

The crystalline phase of BIT piezoelectric ceramics was characterized via X-ray diffraction (XRD) analysis, and the lattice parameters and unit cell volume were refined using the Rietveld method. The cross-sectional morphology, including the presence of a lamellar structure, was examined using a field emission scanning electron microscope (FE-SEM) (Hitachi Corp., Tokyo, Japan). Grain size was quantified using NanoMeasure software based on the SEM images. X-ray photoelectron spectroscopy (XPS) was employed to analyze the chemical composition, particularly to assess oxygen vacancy concentrations, which were calculated using Avantage (v6.9.0) software. The bulk density of the ceramics was measured using the Archimedes water displacement method. To ensure accuracy, each measurement was repeated three times, and the average value was reported. The dielectric constant was measured using an LCR meter, while the piezoelectric coefficient ($d_{33}$) at various sintering temperatures was determined using a piezoelectric coefficient tester.

To more accurately characterize the effect of oxygen vacancy concentration on the electrical properties and microstructure of BIT ceramics, oxygen vacancy concentrations of 18.56%, 29.32%, 32.62%, and 44.55% were calculated using the Vienna Ab initio Simulation Package (VASP). The structural models were constructed using Materials Studio (v2023), and a stochastic method was employed to simulate oxygen vacancies by randomly removing oxygen atoms corresponding to the target concentrations. This study utilized density functional theory (DFT) with projector augmented wave (PAW) pseudopotentials under the Perdew–Burke–Ernzerhof (PBE) generalized gradient approximation (GGA) framework. Ab initio simulations were conducted using VASP (v6.3.2) to analyze the electronic structure, dielectric properties, and ferroelectric polarization of the BIT ceramics. The primary goal was to provide theoretical insights into the enhancement of ferroelectric performance. While the GGA-PBE functional is commonly used to describe delocalized electronic states in most systems, it is inadequate for modeling the localized states present in complex oxides containing Bi and Ti due to strong electron correlation effects. To overcome this limitation, the DFT+U method was adopted to account for on-site Coulomb interactions, resulting in improved agreement with experimental data.

## 3. Results and Discussion

**Figure 1:**
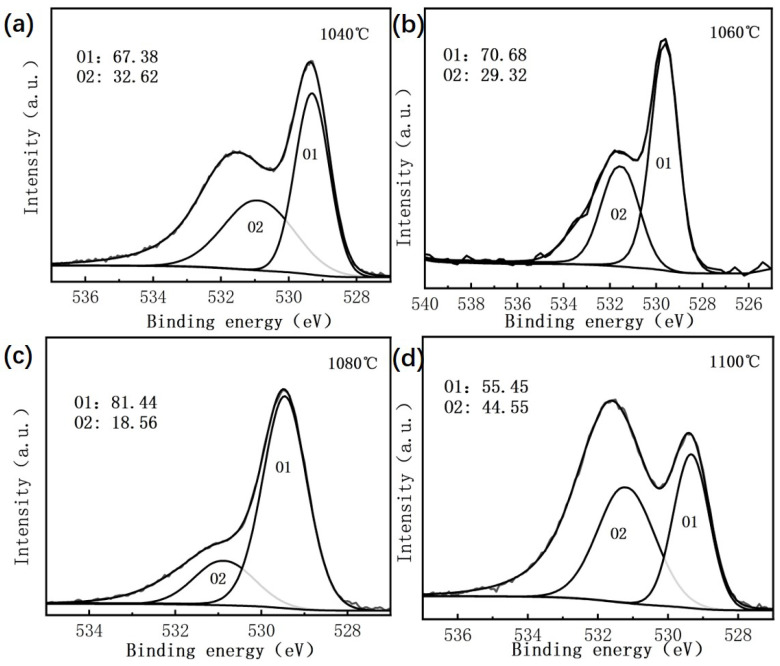
X-ray photoelectron spectroscopy (XPS) of O elements in BIT ceramics at different sintering temperatures. (**a**) 1040 °C (**b**) 1060 °C (**c**) 1080 °C (**d**) 1100 °C.

Figure 1 shows the X-ray photoelectron spectroscopy (XPS) results of BIT ceramics, offering insights into the chemical states of oxygen in the material. As illustrated in Figure 1a–d, the O 1s XPS spectra exhibit two distinct peaks, which can be attributed to variations in electron density and local bonding environments of oxygen atoms in different chemical states, resulting in differences in binding energies [16]. Lattice oxygen and oxygen vacancies correspond to chemically distinct environments, producing separate peaks in the XPS spectra [17]. The lattice oxygen ions (OLattice) are associated with the O1 peak, which appears at a lower binding energy, while the non-lattice oxygen species (ONon-lattice), including hydroxide ions and adsorbed oxygen, are responsible for the O2 peak, observed at higher binding energies [18]. The O2 peak arises due to deviations from stoichiometry in oxygen-deficient regions and is closely related to the presence of oxygen vacancies [19]. The relative proportions of the O1 and O2 peaks are presented in Figure 1.

As the sintering temperature increases from 1040 °C to 1100 °C, the oxygen vacancy concentration in BIT ceramics shows a nonlinear trend, initially decreasing and then increasing. Specifically, the oxygen vacancy concentration is highest at 1040 °C (32.62%), then decreases to 29.32% at 1060 °C and 18.56% at 1080 °C, reaching its lowest value at 1080 °C. At 1100 °C, however, the concentration rises sharply to 44.55%, which is significantly higher than at the lower temperatures. This behavior is attributed to the interplay between the crystal structure and defect dynamics during the sintering process. At the lower temperature of 1040 °C, incomplete sintering leads to the volatilization of $Bi^{3+}$ and $O^{2-}$ ions, resulting in an increased number of oxygen vacancies and reduced crystal ordering. As the sintering temperature increases to 1060 °C and 1080 °C, improved sintering promotes grain densification and defect recovery, thereby filling some oxygen vacancies and resulting in a more complete lattice structure. However, at 1100 °C, the excessively high temperature causes substantial $Bi^{3+}$ volatilization, leading to a cation deficiency at the A-site. This triggers the formation of numerous oxygen vacancies to maintain charge balance. Furthermore, the weakening of grain boundary constraints and the buildup of local stress at high temperatures promote defect formation, causing a dramatic rise in oxygen vacancy concentration. These results clearly demonstrate that sintering temperature plays a critical role in regulating oxygen vacancies in BIT ceramics. An optimal crystal structure with minimal defects and high lattice ordering is achieved at 1080 °C.

**Figure 2:**
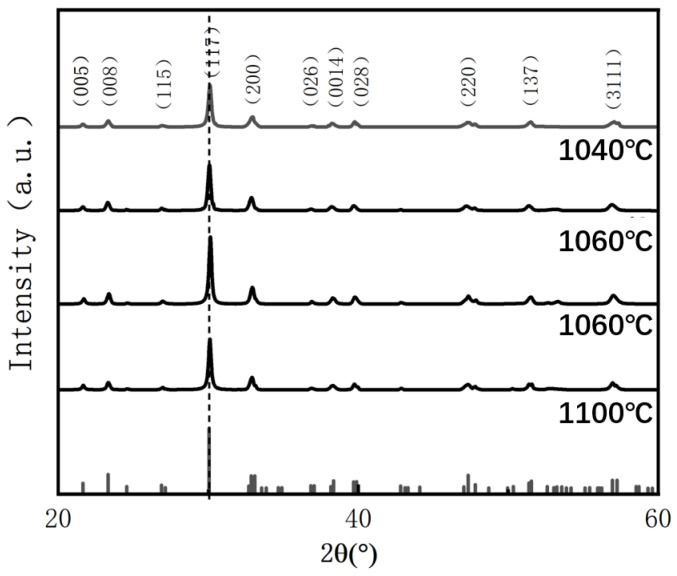
The room-temperature XRD patterns showing the peak intensity relative to BTWO ceramics in the 2θ range 20°–60°.

Figure 2 presents the X-ray diffraction (XRD) patterns of BIT ceramics sintered at various temperatures (measured in the $20^{{}^{\circ}}$–$60^{{}^{\circ}}$ range). As shown, all diffraction peaks correspond to the pure Aurivillius phase (JCPDS No. 72-1019), indicating that no secondary phases are present. This confirms that the samples underwent complete reactions, and no significant impurities formed within the studied sintering temperature range. These findings demonstrate the high phase purity and excellent crystalline integrity of the BIT ceramics. The rhombohedral structure observed is consistent with that of typical bismuth layer-structured ferroelectrics (BLSFs), with the most intense diffraction peaks observed for the (117) crystal plane [20]. This corresponds to the characteristic (112n + 1) diffraction pattern of the BLSF crystal structure, where n represents the number of perovskite-like octahedral layers (n = 3 for BIT) [21]. The intensity of these diffraction peaks serves as an indicator of the crystallographic order and the presence of internal structural defects.

It is noteworthy that the intensity of the strongest diffraction peak initially increases and then decreases with rising sintering temperature, reaching its maximum value (706) at an oxygen vacancy concentration of 18.56%. This trend is primarily attributed to the influence of oxygen vacancies on lattice distortion and structural order. At higher oxygen vacancy concentrations (29.32% and 32.62%), severe lattice distortion occurs, resulting in increased local structural disorder and reduced diffraction intensity on the crystal surface. In contrast, when the oxygen vacancy concentration decreases to 18.56%, an optimal distribution of vacancies is achieved, which promotes lattice relaxation and alleviates internal stresses. This leads to a notable enhancement in the intensity of the (117) diffraction peak due to improved structural ordering [22]. However, when the oxygen vacancy concentration rises to 44.55%, the excessive accumulation of vacancies induces significant lattice distortion and defect clustering, which degrades crystal quality and reduces the diffraction peak intensity. Moreover, variations in oxygen vacancy concentration can alter the interactions between Bi–O layers, thereby affecting the structural stability of the layered system and influencing the material’s diffraction behavior.

**Figure 3:**
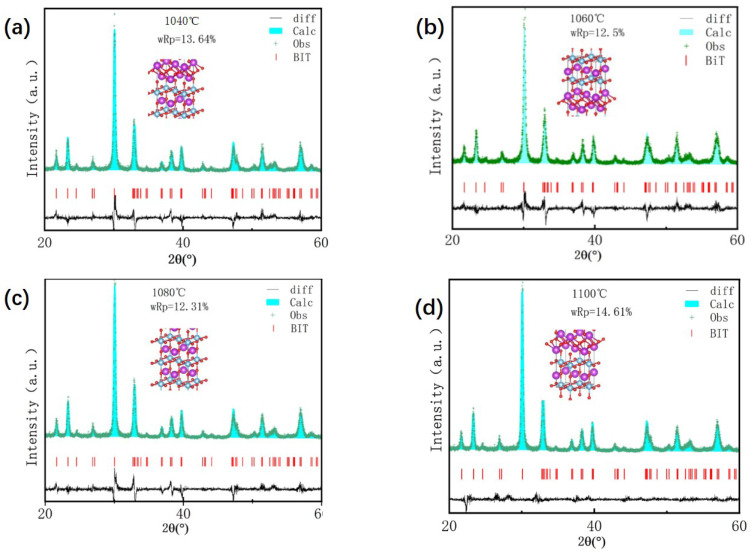
Rietveld refinement data for the room-temperature XRD patterns. (**a**) 1040 °C (**b**) 1060 °C (**c**) 1080 °C (**d**) 1100 °C.

To investigate the crystal parameters, the XRD patterns were analyzed using the Rietveld refinement method (Figure 3), and the refined lattice parameters are summarized in Table 1. As shown in Table 1, variations in the lattice parameters (a, b, c) and unit cell volume, caused by changes in oxygen vacancy concentration with sintering temperature, clearly demonstrate the impact of oxygen vacancies on grain size and lattice distortion in BIT ceramics. The results indicate that as the oxygen vacancy concentration increases, crystal symmetry gradually decreases, accompanied by enhanced lattice distortion. This distortion is most severe at an oxygen vacancy concentration of 44.55%. Furthermore, Table 1 shows that the unit cell volume initially increases and then decreases with increasing oxygen vacancy concentration, reaching a maximum at 29.32%.

**Figure 4:**
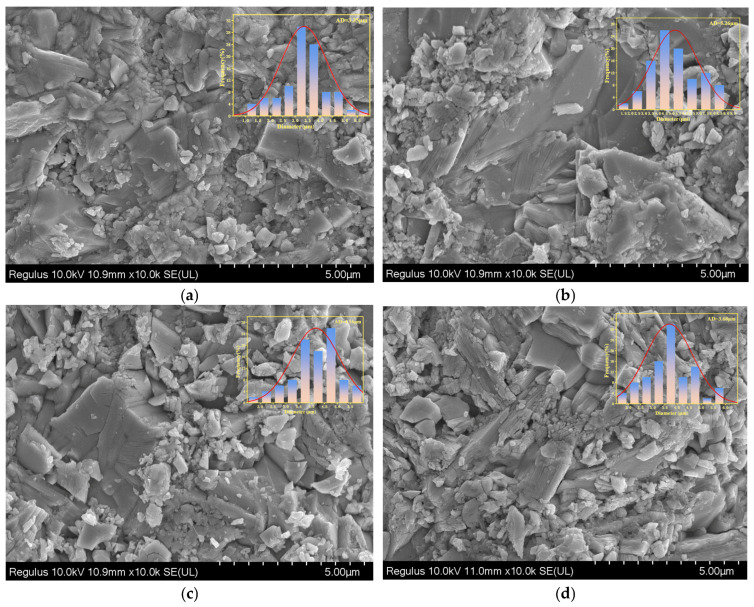
SEM images of BIT ceramics sintered at different temperatures at room temperature. (**a**) 1040 C (**b**) 1060 °C (**c**) 1080 °C (**d**) 1100 °C.

To further investigate the effect of sintering temperature on grain size, scanning electron microscopy (SEM) was employed to analyze the surface morphology of BIT ceramics sintered at different temperatures. Figure 4 presents SEM images showing the microstructure and grain size distribution of BIT ceramic samples at various sintering temperatures. Due to the anisotropic nature of these materials, all samples exhibit typical BLSF crystal structures with plate-like grains. This anisotropy arises from a significantly faster growth rate in the ab-plane than along the c-axis during the early stages of grain growth [23]. As shown in Figure 4, the grain size is relatively uniform, and the samples display high density without apparent pores or cracks. The SEM images were analyzed using NanoMeasure software, and the measured grain sizes at different sintering temperatures were 3.35 μm (1040 °C), 6.26 μm (1060 °C), 4.16 μm (1080 °C), and 3.68 μm (1100 °C), respectively. The grain size of BIT ceramics exhibits a non-monotonic trend with changing oxygen vacancy concentrations (32.62%, 29.32%, 18.56%, 44.55%). This behavior suggests that grain growth is governed not only by oxygen vacancy concentration but also by multiple interacting factors [24].

This phenomenon can be attributed to the fact that a high oxygen vacancy concentration (e.g., 44.55%) enhances grain boundary diffusion, thereby suppressing excessive grain growth and resulting in a reduced grain size of 3.68 μm. Conversely, at a lower oxygen vacancy concentration (e.g., 18.56%), the grain size (4.16 μm) is smaller than that at 29.32% (6.26 μm) due to limited diffusion, which inhibits grain growth. Moreover, a moderate oxygen vacancy concentration (e.g., 29.32%) creates a favorable diffusion environment that promotes sufficient grain growth, yielding the largest grain size (6.26 μm). In comparison, at a relatively high oxygen vacancy concentration (32.62%), the grain size (3.35 μm) is reduced due to the lower initial sintering temperature, which limits grain growth.

**Figure 5:**
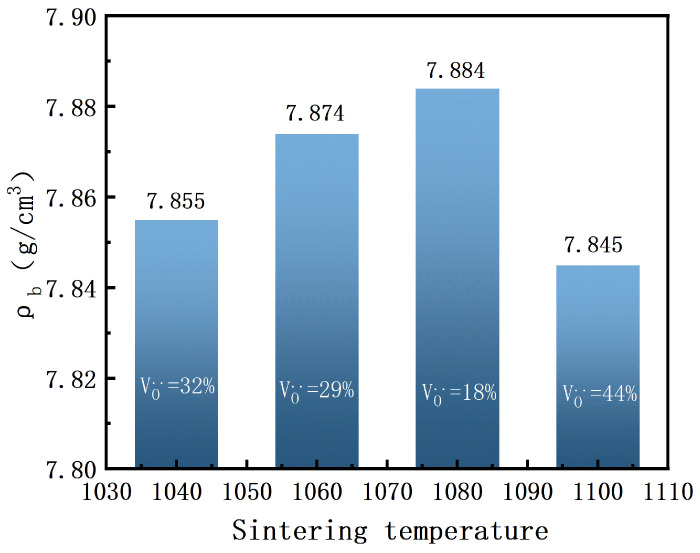
Bulk density of BIT ceramics with different sintering temperatures.

As shown in Figure 5, the bulk density of BIT ceramics is presented as a function of sintering temperature. As shown in Equation (1), the volumetric density of the piezoelectric ceramic cylinder is calculated us ing the Archimedes displacement method. Here, M1 represents the mass of the piezoelectric ceramic cylinder in air, M2 represents the mass of the ceramic cylinder in water, and $\rho_{water}$ denotes the water density corresponding to different temperatures. The calculation is repeated three times, and the average value is taken. The measured bulk densities are 7.845 g/cm^3^, 7.855 g/cm^3^, 7.884 g/cm^3^, and 7.874 g/cm^3^, respectively. Notably, the maximum bulk density (7.884 g/cm^3^) is achieved at a sintering temperature of 1080 °C. This can be attributed to the uniform grain size, low oxygen vacancy concentration, and a notably lower porosity at this temperature, leading to optimal densification [25]. This outcome aligns with the SEM image in Figure 4, indicating that a uniform grain size distribution significantly enhances material densification by minimizing internal pores and grain boundary defects. As a result, the mechanical strength and structural stability of the material are improved. Furthermore, the formation of a dense microstructure inhibits conductive pathways along grain boundaries, thereby reducing electrical conductivity and enhancing the electrical stability and reliability of the material under high-temperature conditions. These findings suggest that a lower oxygen vacancy concentration effectively reduces structural defects, thus improving the overall densification of BIT ceramics.

(1) $$\begin{array}{c} \rho_{ceramic}=M1/\left(M1-M2\right)\rho_{water} \end{array}$$

**Figure 6:**
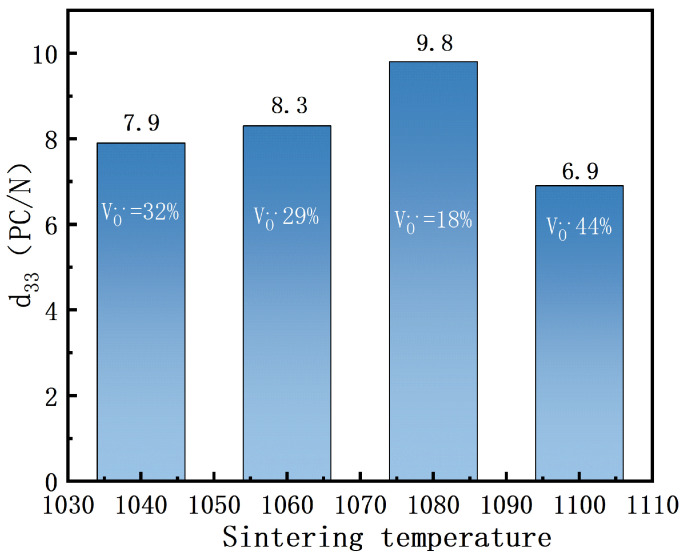
Piezoelectric constants of BIT ceramics with different sintering temperatures.

As shown in Figure 6, the piezoelectric coefficients of BIT ceramics at different sintering temperatures are 7.9 pC/N, 8.3 pC/N, 9.8 pC/N, and 6.9 pC/N, respectively. The highest piezoelectric coefficient (9.8 pC/N) was observed at a sintering temperature of 1080 °C, corresponding to an oxygen vacancy concentration of 18.56%. The relationship between oxygen vacancy concentration and piezoelectric coefficient exhibits a non-monotonic trend. A gradual increase in the piezoelectric coefficient was observed as the oxygen vacancy concentration decreased from 32.62% to 29.32%, indicating that a controlled reduction of oxygen vacancies can enhance piezoelectric properties [26]. The piezoelectric coefficient was further significantly enhanced at the oxygen vacancy concentration of 18.56%, suggesting that this concentration stabilizes the crystal structure and improves the piezoelectric response.In contrast, increasing the oxygen vacancy concentration to 44.55% led to a pronounced decline in the piezoelectric coefficient, indicating that excessive oxygen vacancies cause structural distortion and deteriorate piezoelectric performance. Overall, the effect of oxygen vacancy concentration on piezoelectric properties follows a non-monotonic pattern, where moderate oxygen vacancy concentrations optimize the piezoelectric performance, while excessively high or low concentrations impair the material’s properties.

**Figure 7:**
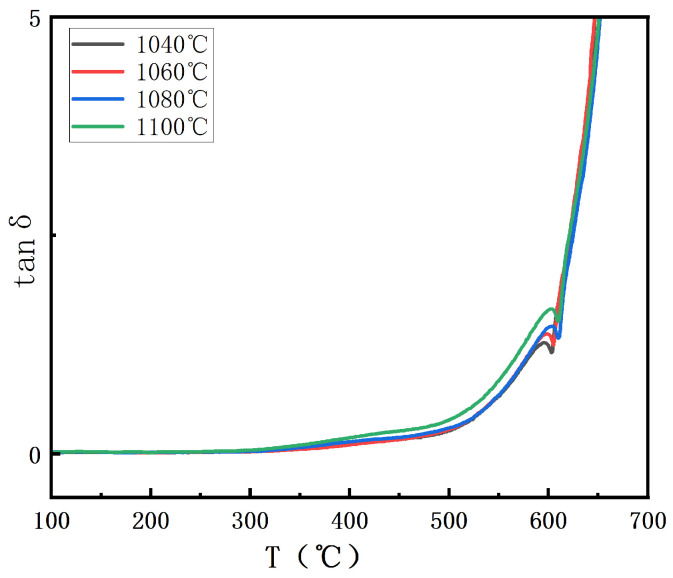
Dielectric loss of BIT ceramics at different sintering temperatures in the range of 100–700 °C.

In Figure 7, the dielectric loss of BIT ceramics at different sintering temperatures is presented. With increasing sintering temperature, the dielectric loss first decreases and then increases. This phenomenon can be attributed to the observation that as the sintering temperature increases, the oxygen vacancy concentration initially decreases and then increases, following a trend consistent with the change in dielectric loss [26]. At high oxygen vacancy concentrations, oxygen vacancies cause an uneven charge distribution within the crystal, leading to a change in the local electric field. This increases the dielectric loss by converting some of the energy into heat [27]. It is evident that when the oxygen vacancy concentration is reduced to 18.56%, the dielectric loss begins to decrease, indicating that the dielectric properties of the material improve significantly at a moderate oxygen vacancy concentration. At this point, the crystal structure is more stable, and charge conduction is more uniform, reducing energy loss and minimizing dielectric loss. However, when the oxygen vacancy concentration increases to 44.55%, dielectric loss increases. The elevated oxygen vacancy concentration enhances the electrical conductivity of the material, which in turn increases dielectric loss. Furthermore, an excess of oxygen vacancies leads to more defect formation within the crystals, creating additional pathways for charge dissipation, thereby further increasing dielectric loss. Therefore, it can be concluded that careful control of oxygen vacancy concentration is essential to optimize the dielectric properties of BIT ceramics.

**Figure 8:**
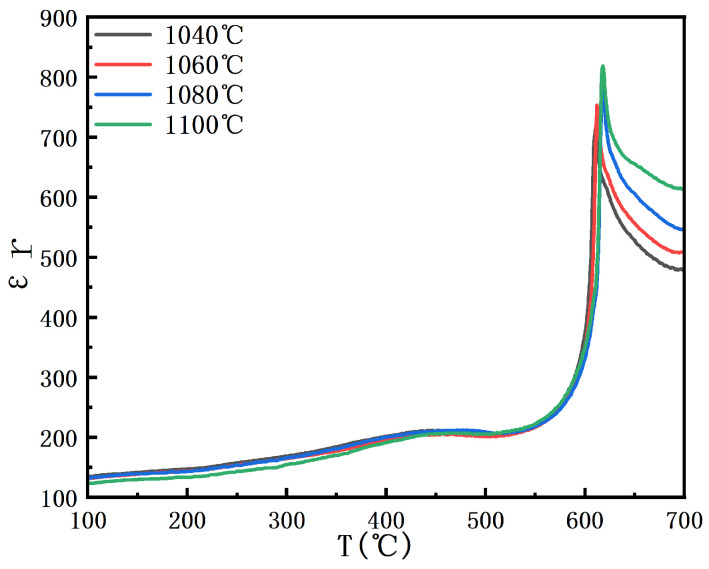
The dielectric constant of BIT ceramics at different sintering temperatures in the range of 100–700 °C.

As shown in Figure 8, the variation in the dielectric constant of BIT ceramics from 100 to 700 °C is presented. The figure shows that the Curie temperatures at sintering temperatures of 1040 °C, 1060 °C, 1080 °C, and 1100 °C are 615 °C, 618 °C, 620 °C, and 612 °C, respectively [28]. The Curie temperature increases and then decreases with changes in grain size, which is closely related to the grain size effect and the resulting variation in densification. As shown in Figure 4, a strong correlation exists between grain size and oxygen vacancy concentration. When the oxygen vacancy concentration is reduced to 18.56%, the grain size exhibits uniformity, and the corresponding bulk density is presented in Figure 4. As the grain size increases, the surface-to-grain-boundary ratio decreases, enhancing long-range ordering within the grains while suppressing surface energy and grain boundary effects [29]. This stabilization of the ferroelectric phase transition ultimately leads to an increase in the Curie temperature. As the grain size increases beyond a certain threshold, defects and internal stresses within grains decrease, accompanied by reduced inter-grain homogeneity. These changes weaken the polarization effect, ultimately leading to a decrease in the Curie temperature. Furthermore, larger grains modify the physical properties of the grain boundary, which further affects the ferroelectric properties of the material. Grain growth initially enhances ferroelectric ordering and increases the Curie temperature. However, once the grain size exceeds a certain threshold, structural degradation and weakened polarization response lower the Curie temperature.

**Figure 9:**
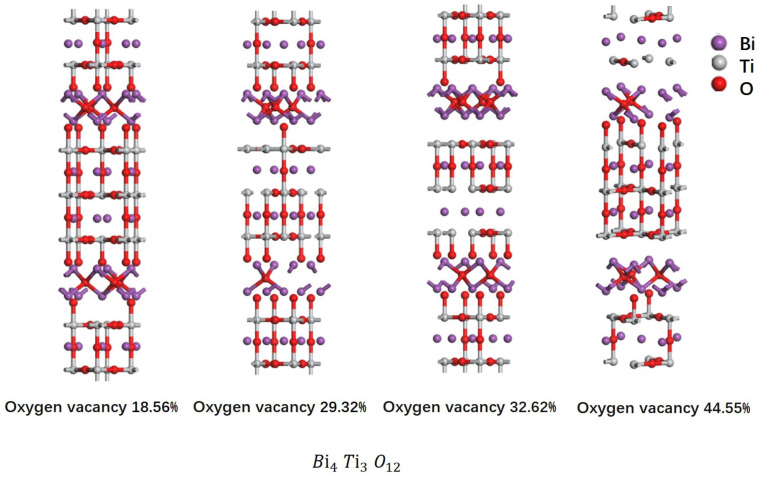
MS modeling of BIT ceramics with different oxygen vacancy concentrations.

To further analyze the effect of oxygen vacancy concentration on the electrical properties, the oxygen vacancy concentration in BIT piezoelectric ceramics was computed using VASP and modeled in MS. The modeling results are presented in Figure 9. The oxygen vacancy concentrations obtained from simulations by randomly removing O atoms using Python (v3.8) were 32.62%, 29.32%, 18.56%, and 44.55%, respectively [29,30]. The modeled data were then subjected to two rounds of structural optimization to minimize errors [31]. Subsequently, the structurally optimized data were used for static calculations. Finally, the results were analyzed through density of states and band structure calculations [32]. The key parameters used in this study include a 500 eV cutoff energy, a Monkhorst–Pack 5 × 5 × 1 k-point grid, and an energy convergence criterion of $10^{-6}$ eV/atom. These parameters ensure the convergence and reliability of the calculations. The computational approach accurately characterizes the density of states (DOS) and band structure of BIT ceramics, thus offering theoretical insights into the effect of oxygen vacancies on the electrical properties of the material.

**Figure 10:**
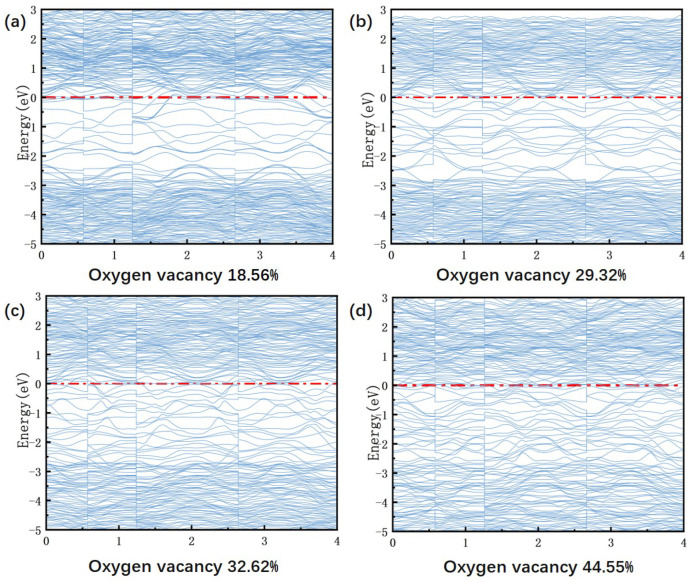
Band structure of BIT ceramics with different oxygen vacancy concentrations calculated using VASP. (**a**) oxygen vacancy concentrations 18.56% (**b**) oxygen vacancy concentrations 29.32% (**c**) oxygen vacancy concentrations 32.62% (**d**) oxygen vacancy concentrations 44.55%.

In Figure 10, the band structures at different oxygen vacancy concentrations, obtained from VASP calculations, are presented. As the oxygen vacancy concentration increases from 18.56% to 44.55%, the band gap gradually decreases from 2.8 eV until it eventually closes completely, indicating a transition to metallic conductivity. This process increases the density of defect states, introducing localized energy levels that merge with the conduction band, thereby facilitating free electron migration [32]. Concurrently, the Fermi level shifts upward, gradually moving from the valence band maximum into the conduction band. This evolution causes the material to change from weak p-type conductivity to n-type semiconducting behavior and eventually to a metallic state. At a low concentration (18.56%), the direct band gap of 2.8 eV corresponds to high resistivity and low leakage current. At this concentration, the Fermi level is close to the valence band maximum, indicating that the material exhibits p-type conductivity. The insulating properties of the material create a favorable environment for the piezoelectric response, resulting in a peak $d_{33}$ value of 9.8 pC/N at an oxygen vacancy concentration of 18.56%. However, when the oxygen vacancy concentration reaches 29.32%, a localized defect state of 1.2 eV forms within the forbidden band, narrowing the band gap to 1.8 eV. This leads to an increase in conductivity and the onset of leakage current. This change aligns with the experimental trend, where $d_{33}$ decreases from 9.8 pC/N (18.56%) to 8.3 pC/N (29.32%). This indicates that carrier scattering increasingly dominates, hindering domain wall motion. Further increasing the oxygen vacancy concentration to 44.55% effectively closes the band gap (Eg ≈ 1.0 eV), with the Fermi level penetrating deeply into the conduction band. This leads to the transformation of BIT ceramics into an n-type semiconductor, an increase in carrier concentration, and a significant rise in leakage current. Ultimately, this results in a reduction of $d_{33}$ to 6.9 pC/N. This energy band evolution demonstrates the ’threshold effect’ of oxygen vacancies on piezoelectric performance by modulating the carrier transport mechanism. It has been found that when the oxygen vacancy concentration exceeds 20%, leakage current becomes dominant, resulting in a sharp degradation of performance.

**Figure 11:**
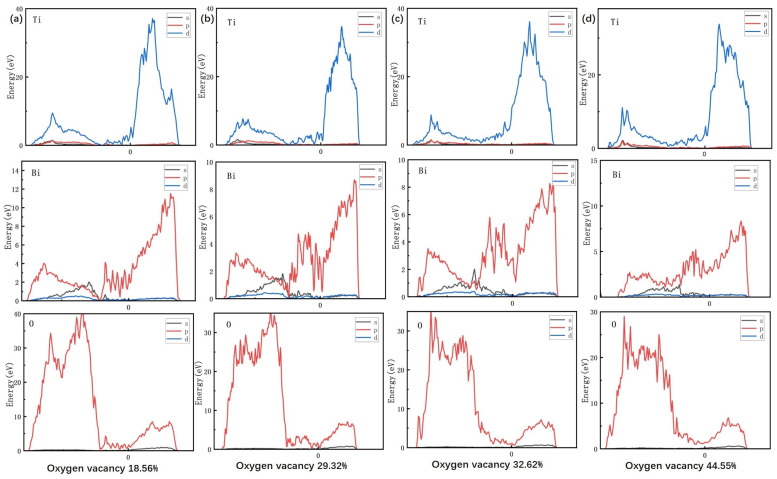
The density of states for Bi, Ti, and O elements is calculated for different oxygen vacancy concentrations using VASP. (**a**) oxygen vacancy concentrations 18.56% (**b**) oxygen vacancy concentrations 29.32% (**c**) oxygen vacancy concentrations 32.62% (**d**) oxygen vacancy concentrations 44.55%.

As shown in Figure 11, the density of states for Bi, Ti, and O elements is calculated for different oxygen vacancy concentrations using VASP. The DOS analysis provides deeper insight into how variations in oxygen vacancy concentration affect the microstructure and macroscopic properties of the material at the atomic scale. At low oxygen vacancy concentrations (18.56%), the density of states of the Ti 3d orbitals is lower near the Fermi energy level (with the main peak ranging from −5 to −3 eV), indicating that Ti is predominantly in the +4 oxidation state ($Ti^{4+}$) and that the strong covalency of the Ti-O bond restricts electron delocalization. At this point, the strong hybridization peaks of the Bi 6p and O 2p orbitals, ranging from −8 to −4 eV and accounting for 65% of the total intensity, indicate the structural integrity of the Bi-O layer. This is consistent with the higher Curie temperature (Tc = 620 °C) and higher piezoelectric coefficient observed in the experiment. As the oxygen vacancy concentration increases to 44.55%, the DOS of the Ti3d orbitals near the Fermi level shows a 120% increase in peak intensity, reflecting a significant increase in the $Ti^{3+}$ ratio. This suggests that oxygen vacancies induce the $Ti^{4+}\to Ti^{3+}$ reduction via electron injection, enhancing local polarization strength. However, the delocalized electrons from excess $Ti^{3+}$ increase the carrier concentration, which directly contributes to the dramatic increase in dielectric loss observed experimentally. Simultaneously, the intensity of the hybridization peaks of Bi 6p with O 2p decreases by 50%, and diffuse O2p defect states emerge within the range of −2 to 0 eV. Oxygen vacancies disrupt the charge balance of the Bi-O layer and weaken the coupled ferroelectric phase transition, resulting in a slight decrease in the Curie temperature from 620 to 618 °C. Charge aggregation near oxygen vacancies forms a localized built-in electric field, enhancing domain flipping (and $d_{33}$) at low concentrations. However, at higher concentrations, this process induces charge disorder and exacerbates lattice distortion. These structural modifications, along with the experimental observation of non-monotonic grain size evolution (most uniformly measuring 4.16 μm at 1080 °C) and fluctuations in bulk density ($7.845\to 7.884\;{g/\mathrm{cm}}^{3}$), suggest the possible impact of oxygen vacancies on microscopic morphology. This supports the idea that dynamic modulation of grain boundary migration and the densification process could be the underlying mechanism.

## 4. Conclusions

In this study, $Bi_{4}Ti_{3}O_{12}$ (BIT) ceramics were prepared using the conventional solid-state reaction method, and their electrical properties were optimized by adjusting the oxygen vacancy concentration. The effect of oxygen vacancies on electrical performance was further analyzed through first-principles calculations using VASP, simulating BIT ceramics with varying vacancy concentrations. The results show that BIT ceramics exhibit a B2cb space group and rhombohedral crystal structure, with a Curie temperature exceeding 610 °C. At a sintering temperature of 1080 °C, the oxygen vacancy concentration is 18.56%, the average grain size is 4.16 μm, the piezoelectric coefficient $d_{33}$ reaches 9.8 pC/N, and the Curie temperature is 620 °C, indicating excellent piezoelectric and thermal stability. VASP calculations reveal that increasing the oxygen vacancy concentration leads to significant changes in both the band gap and carrier transport behavior. Specifically, as the vacancy concentration rises from 18.56% to 44.55%, the band gap decreases from 2.8 eV to approximately 1.0 eV. This transition corresponds to a change from p-type to n-type conductivity, which enhances electrical conductivity but also increases leakage current, consistent with the experimentally observed rise in dielectric loss. This trend also correlates with the reduction in the piezoelectric coefficient $d_{33}$. Density of states (DOS) analysis shows that at low vacancy concentrations, $Ti^{4+}$ is the dominant oxidation state, and the strong covalency of the Ti–O bond limits electron delocalization, which is favorable for high-voltage applications. In contrast, at higher vacancy concentrations, the proportion of $Ti^{3+}$ increases, enhancing electron delocalization and thus increasing conductivity. This behavior aligns with the observed increase in dielectric loss, as well as the decline in both the piezoelectric coefficient and Curie temperature.

This study combines characterization techniques such as XPS, SEM, and XRD with VASP calculation results to investigate the influence of oxygen vacancy concentration on the electrical properties and microstructure of BIT ceramics. The results reveal that tuning oxygen vacancy concentration significantly influences not only carrier transport and densification, but also enhances the electrical performance of BIT ceramics. This study presents a strategy for optimizing the electrical properties of BIT ceramics by modulating the sintering temperature, providing a solid foundation for the development and application of high-performance, high-temperature piezoelectric materials. Moreover, it establishes a theoretical framework for the future utilization of BIT ceramics in high-temperature electronic applications.

## Figures and Tables

**Table 1:** Lattice parameters of $Bi_{4}Ti_{3}O_{12}$ ceramic samples with different sintering temperatures.

Sintering	a (Å)	b (Å)	c (Å)	R-Factor	b/a	Cell	Oxygen
Temperature (°C)						Volume (Å)	Vacancy
1040	5.4578	5.437	32.896	13.64%	0.9955	32.813	32.62%
1060	5.4537	5.4278	32.8699	12.5%	0.9953	32.886	29.32%
1080	5.4517	5.4203	32.868	12.31%	0.9942	32.835	18.56%
1100	5.4541	5.4283	32.857	14.61%	0.9962	32.791	44.55%

## Data Availability

The original contributions presented in this study are included in the article. Further inquiries can be directed to the corresponding author.
